# Genome-wide identification of the PI4P5K gene family in cotton and role of *GhPI4P5K-D04-2* in salt stress tolerance

**DOI:** 10.3389/fpls.2026.1750290

**Published:** 2026-02-25

**Authors:** Cuihan Liu, Qianqian Wang, Zifan Zhao, Yonghang Chen, Han Li, Xuan’ang Wang, Chengwei Li, Eryong Chen

**Affiliations:** 1 Henan Engineering Research Center of Crop Genome Editing, School of Agriculture, Henan Institute of Science and Technology, Xinxiang, China; 2 International Joint Laboratory of Plant Genetic Improvement and Soil Remediation, School of Agriculture, Henan Institute of Science and Technology, Xinxiang, China; 3 College of Life Science and Technology, Xinjiang University, Urumqi, China; 4 School of Agriculture and Biomanufacturing, Zhengzhou University, Zhengzhou, China

**Keywords:** cotton, genome-wide identification, phosphatidylinositol-4-phosphate 5-kinase (PI4P5K/PIP5K) gene family, *GhPI4P5K-D04-2*, salt tolerance

## Abstract

**Introduction:**

Phosphatidylinositol-4-phosphate 5-kinase (PI4P5K/PIP5K), a core regulator of phosphatidylinositol signaling pathways, exerts critical regulatory functions in plant cellular signaling networks and developmental processes, and stress response through its kinase activity. However, its functions in cotton are little reported.

**Methods:**

To comprehensively analyze the *PI4P5K* gene family in cotton, Genome-wide identification was performed to identify cotton PI4P5K family members and analyzed their gene structure, chromosome distribution, systematic evolution and collinearity, and transcript profiles under salt stress. Moreover, we studied function of *GhPI4P5K-D04-2* by transforming it into *Arabidopsis* and using virus-induced gene silencing (VIGS) system.

**Results:**

In this study, we identified 146 PI4P5K family members from four cotton species (*G. arboreum*, *G. raimondii*, *G. barbadense* and *G. hirsutum*) via genome-wide screening, which were phylogenetically divided into three distinct subgroups. Structural domain analysis revealed conserved PIPKc superfamily domain in all proteins, while chromosomal mapping demonstrated syntenic distribution patterns between subgenomes A and D. Integrated transcriptomic and qRT-PCR analyses uncovered *GhPI4P5K-D04-2* as a salt stress-responsive gene. Functional characterization assays demonstrated that overexpressing the *GhPI4P5K-D04-2* gene exhibited enhanced tolerance to salt stress in Arabidopsis, whereas cotton plants with *GhPI4P5K-D04-2* knockdown via VIGS showed increased sensitivity to salt stress.

**Discussion:**

In conclusion, the findings in this study about *PI4P5K* gene family and *GhPI4P5K-D04-2* gene could lay a foundation for future studies of the biological functions of the cotton *PI4P5K* genes, and provide a theoretical basis for targeting improvement of cotton salt resistance through genetic manipulation of PIPK pathway.

## 1 Introduction

As sessile organisms, plants have to adapt to constantly changing environmental conditions throughout their entire life cycle in order to grow better (Ram and Gupta, 1997; Ulukan, 2008). When stimulated by environmental changes, extracellular signals enter the cells via the second messenger system, triggering a series of physiological and biochemical reactions for adaptation (Stevenson et al., 2000). Phosphatidylinositol (PI) and its derivatives have signal-transduction functions in eukaryotic cells. Phosphatidylinositol phosphate kinase (PIPK) is a key enzyme in the phosphatidylinositol signaling pathway, which could catalysis PI monophosphate converting into PI diphosphate (Carafoli, 2002; Hammond and Burke, 2020). The PIPK family is mainly divided into three types: Type I PIPKs or phosphatidylinositol-4-phosphate 5-kinases (PI4P5Ks/PIP5Ks), Type II PIPKs or phosphatidylinositol-5-phosphate 4-kinases (PI5P4Ks/PIP4Ks), and Type III PIPKs or phosphatidylinositol-3-phosphate 5-kinase (PIKfyve/PIPKIII) (Llorente et al., 2023). Animal, yeast, and plant PIPKs share a common basic structure. It is composed of a dimerization domain and a kinase domain highly conserved at the C-terminus (Mueller-Roeber and Pical, 2002). Moreover, a unique conserved domain (the MORN domain) is present at the N-terminus of most plant PIPKs. Compared with animals and fungi, more PIPK genes have been identified in higher plants.

Phosphatidylinositol-4-phosphate 5-kinase (PI4P5K) plays an important role in plant signal transduction and development, with diverse and extensive functions. Whole genome studies have shown that the PI4P5K gene in wheat is associated with high temperature and induces male sterility in anthers (Liu et al., 2021). In tomato, the *PI4P5*K family genes were identified and their expression patterns were analyzed (Wang et al., 2023). The systematic characterization and study of *PI4P5Ks* in plants revealed their functional diversity and evolutionary conservation. In rice, *OsPIP5K1* regulates heading by regulating the expression of flowering inducing genes, and *DWT1/DWL2* works together with *OsPIP5K1* to regulate uniform plant growth (Ma et al., 2004; Fang et al., 2020). In *Arabidopsis*, the *PI4P5K* gene is involved in various stress responses, such as water stress and ABA signaling, as well as osmotic stress adaptation mediated by *PIP5K7*, *PIP5K8*, and *PIP5K9* (Mikami et al., 1998; Kuroda et al., 2021). In addition, *PIP5K7* and *PIP5K9* play a crucial role in the K^+^ efflux triggered by polyamines (Zarza et al., 2020). *PIP5K3* regulates root hair tip growth and PtdIns (3,5) P2 has the function that controls root hair hardening (Kusano et al., 2008; Hirano et al., 2019). Moreover, *PI4P5K* genes also possess other biological functions, such as vacuole morphology, pollen tube growth, and stomatal opening and closing (Lee et al., 2007; Zhao et al., 2010; Ugalde et al., 2016; Hempel et al., 2017). These studies provide important evidence for understanding the role of *PI4P5Ks* in plant growth, development, and stress response, and offer potential targets for crop improvement.

Cotton is an important economic crop and one of the most important fiber and oil crops, planted in more than 80 countries around the world (Chen et al., 2007; Myo et al., 2021). Salt stress is an important factor affecting crop quality and yield. Therefore, improving the salt tolerance of cotton has become an important research topic. This study conducted whole genome identification of cotton PI4P5K family members and analyzed their gene structure, chromosome distribution, systematic evolution and collinearity. Combined with transcriptome data treated with salt stress, PI4P5K genes involved in abiotic stress were identified. Additionally, the *GhPI4P5K-D04–2* gene was identified and its expression changes in response to salt stress were confirmed. The function of the *GhPI4P5K-D04–2* gene under salt stress was also investigated, revealing that it can improve salt tolerance in transgenic *Arabidopsis*. This study enhances our understanding of the cotton *PI4P5K* gene family and provides genetic resources for breeding salt-tolerant cotton.

## 2 Materials and methods

### 2.1 Plant materials and growth conditions

*Arabidopsis thaliana* ecotype Col-0 (WT) served as both the wild-type control and transgenic recipient for *GhPI4P5K-D04–2* overexpression studies. *Arabidopsis* seeds were surface-sterilized with 70% ethanol for 2 min followed by 5% sodium hypochlorite (NaClO) treatment for 15 min, then rinsed thoroughly with sterile water. Three independent overexpression lines were generated through *Agrobacterium tumefaciens*-mediated floral dipping of flower buds at the rosette stage. Seeds from WT and transgenic lines were stratified on MS solid medium containing 0 mM, 125 mM NaCl under 4 °C vernalization for 2 days to synchronize germination. Germinated seedlings were transferred to a controlled growth chamber maintained at 22 °C with 16 h light/8 h dark photoperiod and 60% relative humidity for phenotypic analysis.

Taking the cotton (*Gossypium hirsutum* L.) line CCRI24 obtained from the Cotton Research Institute of the Chinese Academy of Agricultural Sciences as the research object. To analyze the expression of *GhPI4P5K-D04–2* after treatment with NaCl (150 mM), cotton seeds were wrapped in wet filter paper and placed vertically in a water basin containing 1L of water. Place the water basin in a 25 °C incubator under 16 h light/8 h dark photoperiods. After the cotton seeds germinate, they are transferred to Hoagland’s solution for growth until the three-leaf stage. Then, leaf samples were collected after processing 0, 1, 3, 6, 12, and 24 hours under high salt (150 mM NaCl) conditions. The samples are frozen in liquid nitrogen and stored at -80 °C for RNA isolation and cDNA preparation.

### 2.2 Identification of the *PI4P5K* gene family in cotton

The genomic data for *G*. *arboretum* (*G*. *arboreum*_V1.0, CRI, https://www.cottongen.org/species/Gossypium_arboreum/CRI-A2_genome_v1.0), *G*. *raimondii* (*G*.*raimondii*_221_V2.0, JGI, https://www.cottongen.org/species/Gossypium_raimondii/jgi_genome_221), *G*. *barbadense* (Hai7124_V1.1, ZJU, https://www.cottongen.org/data/download/genome_tetraploid/AD2) and *G*. *hirsutum* (TM-1_V2.1, ZJU, https://www.cottongen.org/species/Gossypium_hirsutum/ZJU-AD1_v2.1) were downloaded from the CottonGen (https://www.cottongen.org/), including coding sequences (CDS), gene annotations, and protein sequences. The Pfam number PF01504 of the *PI4P5K* gene family was searched through the Pfam38.0 database (http://pfam-legacy.xfam.org/) (Mistry et al., 2021), and the hidden Markov model (HMM) of the *PI4P5K* gene family was downloaded. The sequences containing PI4P5K protein domains in the protein files of *G*. *hirsutum* were searched via HMMER3.3.2 software (http://www.HMMER.org/) (Potter et al., 2018). The E value was set to 1e-10 to screen candidate protein sequences. The two major non-functional homologues, including pseudogenes and erroneous sequences, are often considered noise that has no significant effect on the results. Therefore, non-functional homologues sequences in gene family identification were removed using seqrutinator with default parameter (Amalfitano et al., 2024). The retained sequences were validated further the conserved domain of PI4P5K protein in NCBI-CDD (https://www.ncbi.nlm.nih.gov/cdd/). Using the same method, we obtained other three predicted cotton *PI4P5K* genes from *G. arboreum*, *G. raimondii*, and *G. barbadense*.

### 2.3 Phylogenetic analysis of the *PI4P5K* gene family

In order to analyze the genetic diversity of the cotton PI4P5K family, multiple sequence alignment of *G*. *arboreum*, *G*. *raimondii, G*. *barbadense* and *G*. *hirsutum* were performed using Clustal W. Phylogenetic trees using the neighbor-joining (NJ) method with the bootstrap value setting 1000 replicates were constructed for 4 cotton species and *G*. *hirsutum* in MEGA7.0 software, respectively.

### 2.4 Gene structure, protein domains and physic-chemical property analysis of the *PI4P5K* gene family

Exon-intron structure representations based on basic coding information were displayed using the online program GSDS2.0 (http://gsds.cbi.pku.edu.cn/). Protein domains of the cotton PI4P5K family were annotated using online SMART software (http://smart.embl-heidelberg.de/), and visualized using Gene Structure View in TBtools software (Liu et al., 2024). Physicochemical properties of PI4P5K proteins were calculated and subcellular localization predictions obtained using ProtParam (https://web.expasy.org/protparam/) and CELLO RESULTS (http://cello.life.nctu.edu.tw/) online software. In addition, the hydrophilicity (GRAVY) of PI4P5K protein was analyzed using ProtParam online tool (http://web.expasy.org/protparam/).

### 2.5 Chromosomal location, collinearity analysis of the *PI4P5K* genes

Chromosome location information of *PI4P5K* gene family members was extracted from upland cotton genome annotation files. The distribution map of the *PI4P5K* genes on the upland cotton chromosomes was drawn in the TBtools software according to their specific physical locations (bp). Collinearity of homologous genes of PI4P5K proteins were analyzed using default parameters by MCScanX (https://github.com/wyp1125/MCScanX), and gene duplication events between the two were visualized using TBtools (Liu et al., 2024).

### 2.6 Analysis of PI4P5K expression profiles using RNA-seq data

Relative expression of 48 upland cotton PI4P5K genes for 11 tissues (Ovule, Fiber, Root, Stem, Leaf, Torus, Petal, Sepal, Epicalyx, Anther, and Pistil) and salt stress with 200 mM NaCl treatment of upland cotton “TM-1” *PI4P5K* genes could be download by website page from the cottonomics database (http://cotton.zju.edu.cn/10.rnasearch.html). The downloaded data have been normalized and averaged by cottonomics (Dai et al., 2022). The expression of upland cotton *PI4P5K* genes under salt stress at 0 h, 1 h, 3 h, 6 h, 12 h and 24 h was analyzed and a heatmap will be drawn using TBtools software (Liu et al., 2024).

### 2.7 RT-PCR and quantitative real-time PCR

RNA was extracted by RNA isolation kit (Cat # DP441, Tiangen, Beijing, China) after samples were collected, and cDNA were synthesized using cDNA synthesis kit (Cat # RR037A, TaKaRa, Dalian, China). The RT-PCR and qRT PCR methods are based on our previous research (Chen et al., 2021). RT-PCR and qRT-PCR were performed using *AtUBQ10* and *GhHIS3* as internal references, respectively.

### 2.8 Cloning of *GhPI4P5K-D04–2* gene and transformation into *Arabidopsis*

The *GhPI4P5K-D04–2* gene coding sequence was amplified by specific primers and inserted into p6MYC to generate an overexpression vector. The restriction sites used in the vector were Kpn I/Sac I. *Arabidopsis* transformation is carried out through the flower soaking method (Clough and Bent, 1998). Transgenic plants were select on MS solid plates containing 50 µg/L kanamycin. The primers used for cloning *GhPI4P5K-D04–2* in this study are as follows: forward (F) (5′- GGTACCTATGTCTGGCCCCGTGGTC-3′) and reverse (R) (5′- GAGCTCTCAACCTTTAATGGAGTTTTGA -3′).

### 2.9 VIGS of *GhPI4P5K-D04–2* and NaCl treatment

The tobacco rattle virus (TRV2:00) plasmid was digested with restriction enzyme *Sac* I and *Xba* I, which were combined with the target fragment to generate TRV2:*GhPI4P5K-D04–2* by specifically designed primers. Subsequently, the correctly sequenced TRV2:*GhPI4P5K-D04–2* plasmid was transformed into *Agrobacterium tumefaciens* GV3101, was mixed with auxiliary bacteria, and was injected into two fully unfolded cotton seedlings. Salt treatment (200 mM) was performed on cotton at the three leaf stage, and the silencing efficiency was confirmed by qRT-PCR experiments. The specific primers for constructing the *GhPI4P5K-D04–2* VIGS vector are as follows: forward (F) (5′- TCTAGAATGTCTGGCCCCGTGGTC-3′) and reverse (R) (5′- GAGCTCAGAGTCAATATATGTCCCAGTA -3′). Three biological replicates were analyzed.

## 3 Results

### 3.1 Phylogenetic analysis of the PI4P5K proteins in cotton

**Figure 1:**
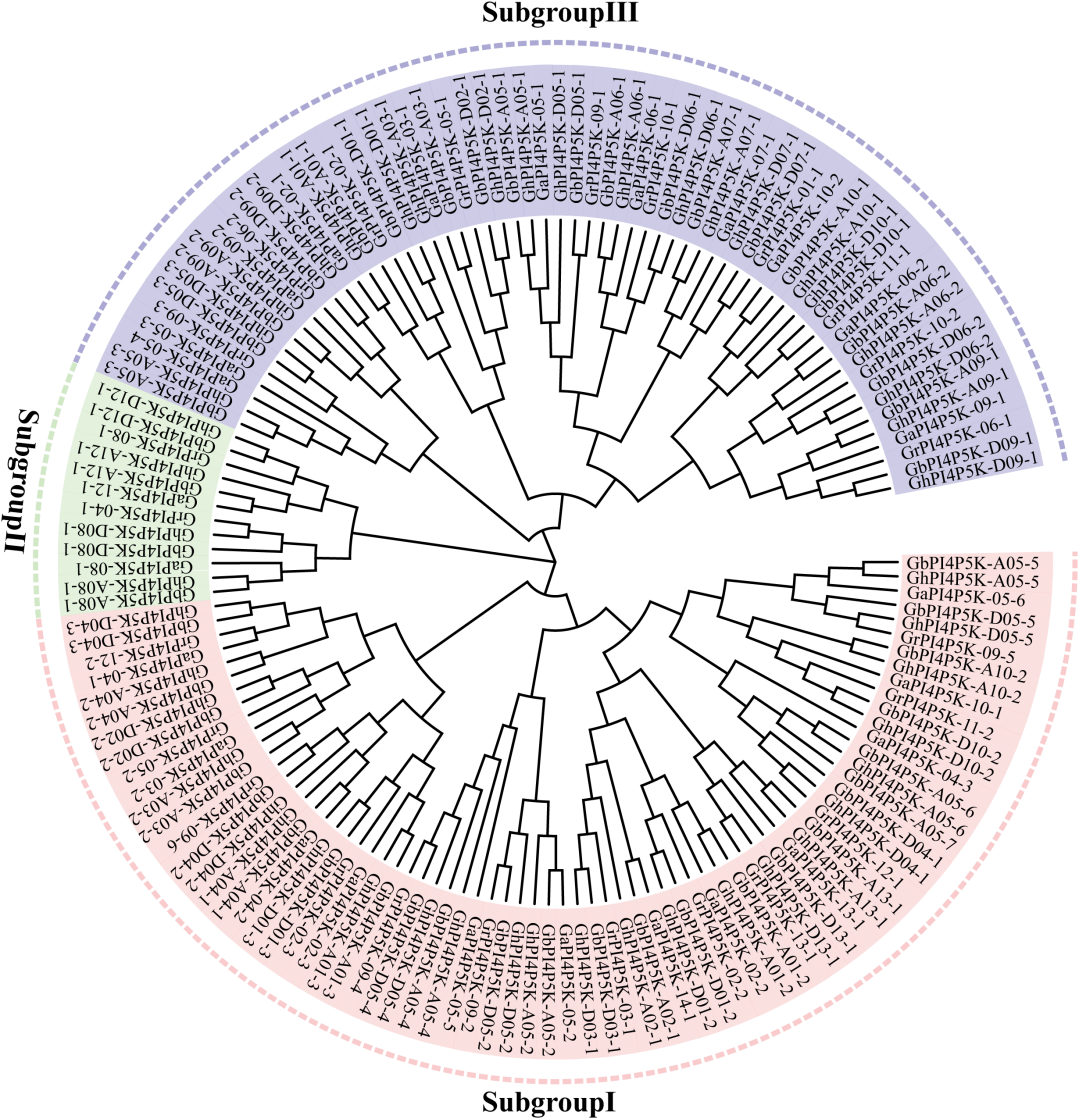
Phylogenetic relationships of PI4P5K family members in four cotton species. Phylogenetic relationship analysis of PI4P5K proteins from *G. arboreum*, *G. raimondii*, *G. barbadense* and *G. hirsutum*. The phylogenetic tree was constructed by the neighbor-joining (NJ) method using MEGA 7.0. Bootstrap values were 1000 repeats.

We identified putative *PI4P5K* genes in four cotton species. A total of 146 *PI4P5K* genes were identified, including 25 in *G. arboreum*, 24 in *G. raimondii*, 49 in *G. barbadense* and 48 in *G. hirsutum* (**Supplementary Table S1**). To understand the evolutionary relationship of the PI4P5K protein family, a phylogenetic tree was constructed using the PI4P5K protein amino acid sequences from *G. arboreum*, *G. raimondii*, *G. barbadense* and *G. hirsutum* by the NJ method of MEGA7.0 software. The results showed that all the PI4P5K proteins can be divided into three subgroups: subgroup I, subgroup II, and subgroup III (**Figure 1**). The number of *PI4P5K* genes in *G. arboreum* and *G. raimondii* in each subgroup was basically half the number in *G. hirsutum* and *G. barbadense* in each subgroup. The results indicated that the evolution of *PI4P5K* genes in cotton were relatively conserved. Moreover, the subgroup I has the largest members of *PI4P5K* genes, while the subgroup II contains fewest *PI4P5K* gene members in cotton.

### 3.2 Phylogenic tree, protein structural domain and gene structure analysis

**Figure 2:**
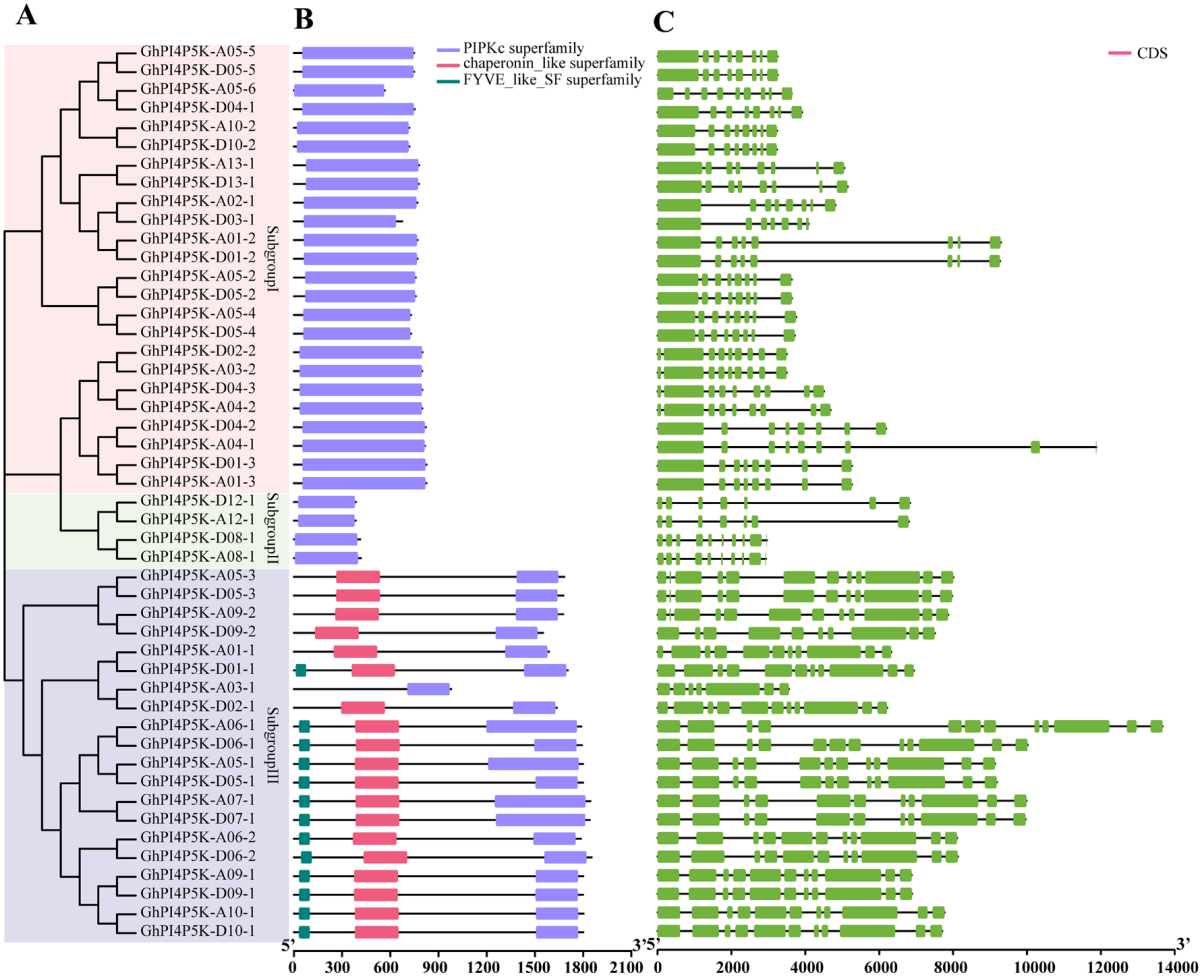
Analysis of phylogenetic tree, conserved domains and gene structure of PI4P5K family members in (*G*) *hirsutum*. **(A)** The phylogenetic tree of GhPI4P5K proteins. The tree was constructed with a bootstrap of 1000 by the neighbor-joining (NJ) method in MEGA. **(B)** Conserved domains of GhPI4P5K proteins. The purple/red/blackish green rectangles represent the PIPKc superfamily domain, chaperonin-like superfamily domain, and FYVE-like-SF superfamily domain, respectively. The size of proteins can be estimated using the bottom scale. **(C)** The gene structure of *GhPI4P5K* genes. Exons and introns are shown with light green boxes and black lines, respectively. The sizes of exons and introns can be estimated using the scale at the bottom.

In order to gain a deeper understanding of the basic information of the PI4P5K family in cotton, we compared the full-length protein sequences of *PI4P5K* genes from four cotton species (*G. arboreum*, *G. raimondii*, *G. barbadense* and *G. hirsutum*) and constructed a phylogenetic tree containing conserved domains, and exons and introns of the genes (**Supplementary Figure S1**). Specially, we focused on analyzing the domains and gene structure of PI4P5Ks in *G. hirsutum* species (**Figure 2**). The result showed that three types of conserved domains were identified in PI4P5K protein family of *G. hirsutum*, and all proteins contain PIPKc superfamily domain, making it the core domain of the GhPI4P5K family (**Figure 2B**). What’s more, only one domain could be found in GhPI4P5K proteins of subgroup I and subgroup II. While, in subgroup III, one GhPI4P5K protein has one domain, six GhPI4P5K proteins each contain two domains, and thirteen GhPI4P5K proteins each possess three domains (**Figure 2B**). These results suggest that GhPI4P5K proteins of subgroup III might have a more important and specific functions in cotton growth and development. The structural diversity was explored to gain insights into the structural evolution of the *GhPI4P5K* genes. The results revealed that the *GhPI4P5K* genes contain exon numbers ranging from 7 to 12, and the intron numbers ranging from 6 to 11 (**Figure 2C**). Moreover, within the same subgroup, most members have significant similarities in gene structure (**Figure 2C**).

Further, the physicochemical properties of all the upland cotton PI4P5K proteins were analyzed, and the results showed that all the upland cotton PI4P5K proteins were very different in terms of protein length, protein molecular weight (MW) and isoelectric point (pI) (**Table 1**). In upland cotton, PI4P5K proteins had an average length of 1125 amino acids and varied in length from 384 (*GhPI4P5K-D12-1*) to 1853 (*GhPI4P5K-D06-2*) amino acids, with molecular weights varying from 44.35 to 208.63 KDa. The isoelectric point (pI) values of the upland cotton PI4P5K proteins ranged from 5.32 to 9.16. The overall mean of the hydrophilicity (GRAVY) scores for all PI4P5K proteins was negative, indicating that PI4P5K proteins were hydrophilic. Subcellular localization prediction results showed that 24 PI4P5K proteins were displayed in the nuclear and 18 PI4P5K proteins were displayed in the cytoplasm. The remaining six PI4P5K proteins were predicated to localize in the nuclear and cytoplasm, which was consistent with hydrophilic of the overall mean of the GRAVY scores.

**Table 1:** The physicochemical properties of *PI4P5K* genes in *G. hirsutum*.

Gene Name	Transcript ID	Chr.	Start Postion	End Postion	N. Amino Acid	MW(KDa)	pI	GRAVY	Predicted Subcellular Location
GhPI4P5K-A01-1	GH_A01G1648	A01	92002388	92008716	1587	176.80	5.58	-0.425	Nuclear
GhPI4P5K-A01-2	GH_A01G2098	A01	112734031	112743320	770	88.58	8.59	-0.711	Cytoplasmic
GhPI4P5K-A01-3	GH_A01G2293	A01	116154200	116159457	825	93.09	8.93	-0.521	Cytoplasmic, Nuclear
GhPI4P5K-A02-1	GH_A02G1290	A02	55947283	55952100	768	88.79	6.05	-0.697	Cytoplasmic
GhPI4P5K-A03-1	GH_A03G1435	A03	88755135	88758691	978	110.95	7.32	-0.36	Nuclear
GhPI4P5K-A03-2	GH_A03G2365	A03	111080476	111083975	798	90.51	9.06	-0.557	Cytoplasmic
GhPI4P5K-A04-1	GH_A04G0885	A04	64275642	64287521	815	92.00	8.77	-0.491	Cytoplasmic, Nuclear
GhPI4P5K-A04-2	GH_A04G1543	A04	85541790	85546475	799	90.52	8.79	-0.595	Cytoplasmic
GhPI4P5K-A05-1	GH_A05G0370	A05	3520729	3529865	1799	199.79	5.64	-0.522	Nuclear
GhPI4P5K-A05-2	GH_A05G2320	A05	22878122	22881750	758	86.50	8.5	-0.632	Cytoplasmic
GhPI4P5K-A05-3	GH_A05G2495	A05	25913906	25921913	1683	186.55	5.32	-0.428	Nuclear
GhPI4P5K-A05-4	GH_A05G2701	A05	29207715	29211464	729	83.80	8.01	-0.713	Cytoplasmic
GhPI4P5K-A05-5	GH_A05G2841	A05	32106949	32110206	748	85.40	9.02	-0.718	Cytoplasmic, Nuclear
GhPI4P5K-A05-6	GH_A05G4205	A05	108707451	108711101	565	64.45	7.57	-0.589	Cytoplasmic
GhPI4P5K-A06-1	GH_A06G0917	A06	27043151	27056819	1788	197.35	5.75	-0.409	Nuclear
GhPI4P5K-A06-2	GH_A06G1673	A06	112085331	112093437	1786	201.48	5.76	-0.538	Nuclear
GhPI4P5K-A07-1	GH_A07G0181	A07	1828165	1838155	1844	203.77	5.68	-0.467	Nuclear
GhPI4P5K-A08-1	GH_A08G1524	A08	101373179	101376113	416	47.72	8.83	-0.388	Nuclear
GhPI4P5K-A09-1	GH_A09G0943	A09	60154702	60161581	1800	202.07	5.48	-0.49	Nuclear
GhPI4P5K-A09-2	GH_A09G1343	A09	68518521	68526385	1676	187.64	5.55	-0.4	Nuclear
GhPI4P5K-A10-1	GH_A10G0515	A10	4942060	4949831	1802	201.67	5.59	-0.459	Nuclear
GhPI4P5K-A10-2	GH_A10G2027	A10	103301579	103304810	718	81.36	7.8	-0.617	Cytoplasmic
GhPI4P5K-A12-1	GH_A12G1261	A12	80883206	80890040	386	44.71	8.47	-0.232	Nuclear
GhPI4P5K-A13-1	GH_A13G0020	A13	190415	195468	779	89.41	8.37	-0.659	Cytoplasmic
GhPI4P5K-D01-1	GH_D01G1741	D01	48578888	48585826	1704	189.92	5.7	-0.435	Nuclear
GhPI4P5K-D01-2	GH_D01G2192	D01	60171967	60181238	770	88.60	8.41	-0.713	Cytoplasmic
GhPI4P5K-D01-3	GH_D01G2372	D01	62801478	62806736	825	93.05	8.93	-0.53	Cytoplasmic, Nuclear
GhPI4P5K-D02-1	GH_D02G1586	D02	53060329	53066541	1636	184.35	6.25	-0.397	Nuclear
GhPI4P5K-D02-2	GH_D02G2535	D02	69303015	69306514	800	90.63	9.16	-0.575	Cytoplasmic, Nuclear
GhPI4P5K-D03-1	GH_D03G0675	D03	16754211	16758302	673	77.58	5.83	-0.74	Cytoplasmic
GhPI4P5K-D04-1	GH_D04G0173	D04	2209506	2213419	751	85.10	6.72	-0.651	Cytoplasmic
GhPI4P5K-D04-2	GH_D04G1204	D04	40153619	40159803	821	92.89	8.9	-0.505	Cytoplasmic
GhPI4P5K-D04-3	GH_D04G1886	D04	54590151	54594654	799	90.55	8.92	-0.591	Cytoplasmic
GhPI4P5K-D05-1	GH_D05G0375	D05	3025487	3034667	1799	199.45	5.54	-0.519	Nuclear
GhPI4P5K-D05-2	GH_D05G2342	D05	20782989	20786627	758	86.34	8.41	-0.643	Cytoplasmic
GhPI4P5K-D05-3	GH_D05G2507	D05	22988675	22996651	1677	186.03	5.37	-0.428	Nuclear
GhPI4P5K-D05-4	GH_D05G2718	D05	25468763	25472478	729	83.63	8.18	-0.711	Cytoplasmic
GhPI4P5K-D05-5	GH_D05G2857	D05	27471651	27474911	748	85.45	9.02	-0.724	Cytoplasmic, Nuclear
GhPI4P5K-D06-1	GH_D06G0893	D06	16261756	16271773	1792	197.91	5.62	-0.42	Nuclear
GhPI4P5K-D06-2	GH_D06G1687	D06	53797878	53806006	1853	208.63	5.54	-0.523	Nuclear
GhPI4P5K-D07-1	GH_D07G0192	D07	1812331	1822295	1842	203.32	5.62	-0.444	Nuclear
GhPI4P5K-D08-1	GH_D08G1541	D08	50322839	50325794	411	46.98	8.61	-0.39	Nuclear
GhPI4P5K-D09-1	GH_D09G0897	D09	33183954	33190842	1799	201.56	5.51	-0.494	Nuclear
GhPI4P5K-D09-2	GH_D09G1294	D09	39260667	39268172	1550	173.49	5.48	-0.387	Nuclear
GhPI4P5K-D10-1	GH_D10G0541	D10	4638682	4646389	1801	202.01	5.62	-0.46	Nuclear
GhPI4P5K-D10-2	GH_D10G2136	D10	56125869	56129098	718	81.41	7.52	-0.625	Cytoplasmic
GhPI4P5K-D12-1	GH_D12G1277	D12	39945065	39951865	384	44.35	8.91	-0.248	Nuclear
GhPI4P5K-D13-1	GH_D13G0016	D13	161448	166593	777	89.21	8.46	-0.666	Cytoplasmic

### 3.3 Chromosomal location, collinearity analysis of the *PI4P5K* genes

**Figure 3:**
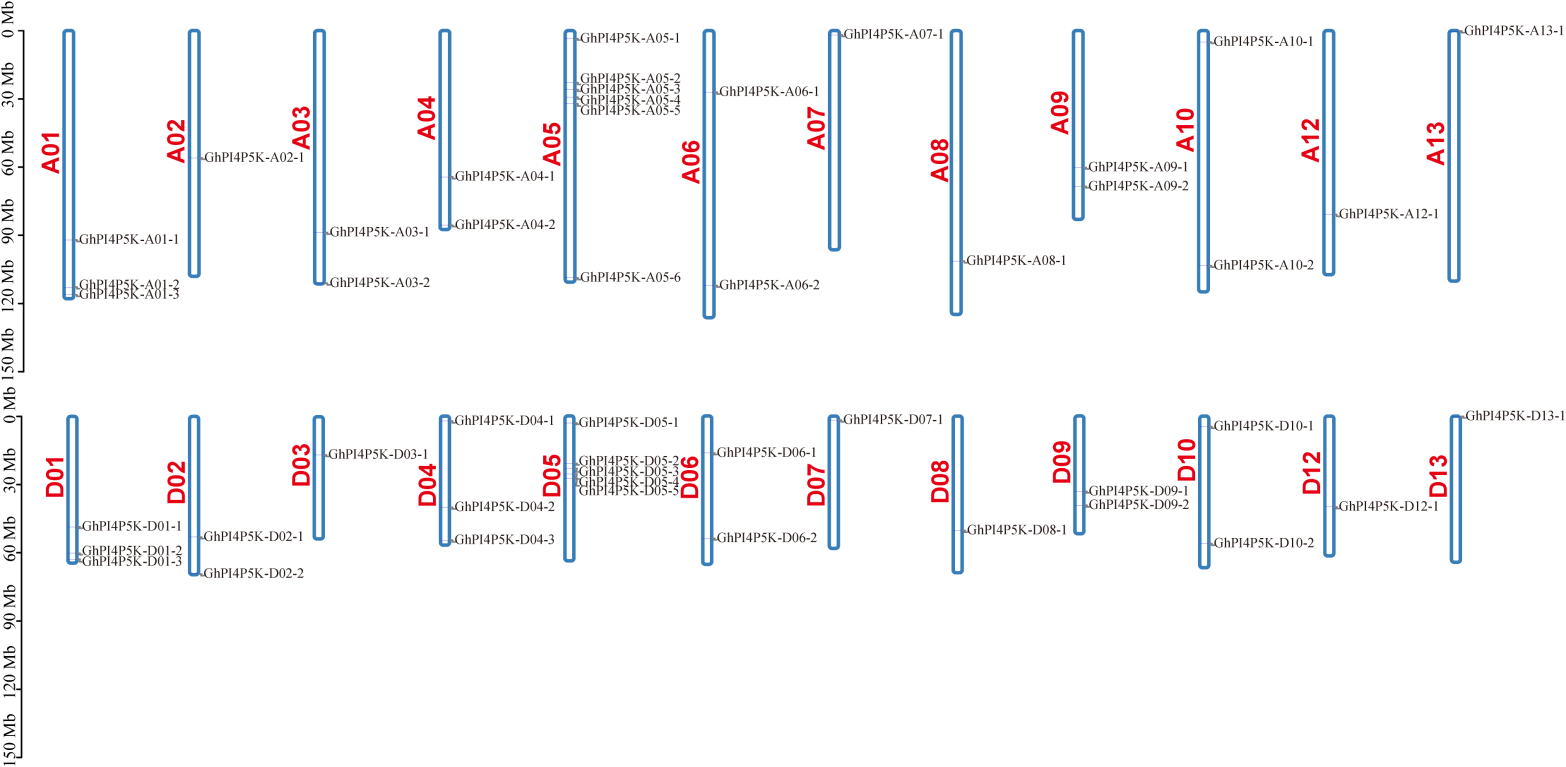
Chromosome locations of the *PI4P5K* genes in *G. hirsutum*.

There was a total of 48 *GhPI4P5K* genes distributed on 24 chromosomes of *G. hirsutum*, of which 24 genes were distributed on 12 chromosomes of the A subgenome and D subgenome, respectively (**Figure 3**). While there were no *GhPI4P5K* genes distributed on A11 and D11 chromosomes. Except the chromosome 02, 03, 04 and 05 of subgenome A and D had different gene numbers, all the other corresponding chromosomes of the two subgenomes contained the same gene numbers, and the highest number of *GhPI4P5K* genes were on chromosome A05 and D05. In addition, observations had shown that the majority of *GhPI4P5K* genes were distributed at both ends of chromosomes. This telomere-proximal distribution might be associated with higher recombination rates and gene density in these regions, which could facilitate gene family expansion and functional diversification. Furthermore, such a distribution pattern may influence the regulation and expression of these genes. Whatever, the *GhPI4P5K* gene distribution on the A and D subgenomes was relatively similar, indicating a strong correlation between the two subgenomes. The chromosomal location results of *GhPI4P5K* genes were in line with the evolutionary relationship of polyploid cotton.

**Figure 4:**
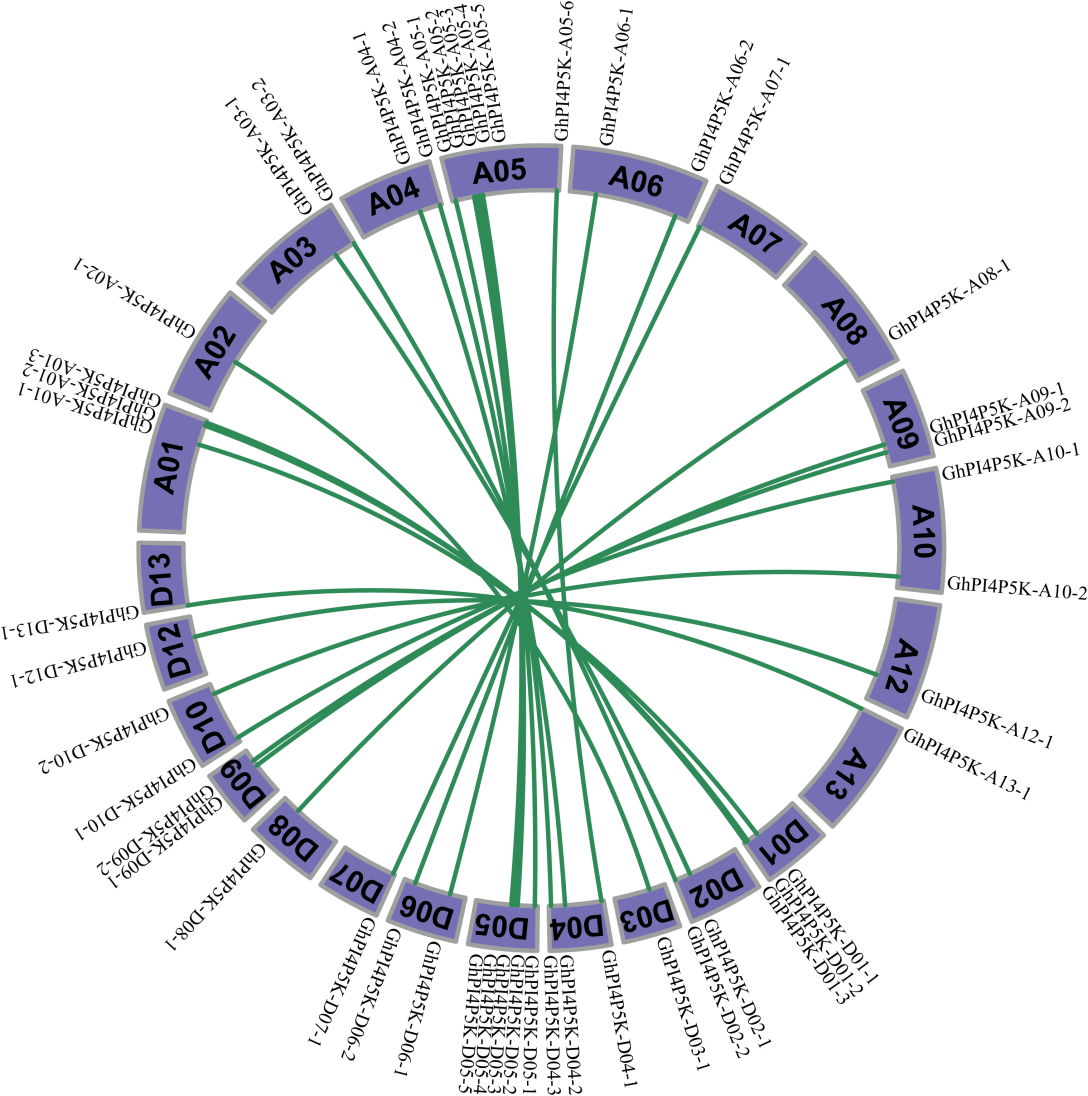
Collinearity relationship analysis of *GhPI4P5K* genes in subgenome A and subgenome D.

We further analyzed the collinearity relationships for all *PI4P5K* genes on the chromosomes At and Dt of *G*. *hirsutum* with other Gossypium species. Results showed that 24 *PI4P5K* genes on A subgenome had intergenomic homologous genes on the chromosomes D subgenome of *G*. *hirsutum*. In-depth studies found that *PI4P5K* genes on chromosomes A01/D01, A04/D04, A05/D05, A06/D06, A07/D07, A08/D08, A09/D09, A10/D10, A12/D12 and A13/D13 exhibited strong one-to-one collinearity (**Figure 4**). Interestingly, genes of *GhPI4P5K-A02-1*, *GhPI4P5K-A03-1*, *GhPI4P5K-A03–2* and *GhPI4P5K-A05–6* exhibited collinearity with *GhPI4P5K-D03-1*, *GhPI4P5K-D02-1*, *GhPI4P5K-D02-2*, and *GhPI4P5K-D04-1*, respectively. To explore the evolutionary relationship of *PI4P5K* genes in cotton, we performed synteny analysis of genome of *G*. *arboreum* and *G*. *hirsutum* A subgenomes, genome of *G*. *raimondii* and *G*. *hirsutum* D subgenome, *G*. *hirsutum* and *G*. *barbadense*. Among 24 *PI4P5K* genes on A and D subgenome had intergenomic homologous genes in *G*. *arboreum* and *G*. *raimondii*, respectively (**Supplementary Figures S2**, **S3**). The 48 *PI4P5K* genes in *G. hirsutum* and the 49 *PI4P5K* genes in *G. barbadense* had intergenomic homologous relationship, including *GhPI4P5K-A05–6* exhibited collinearity with *GbPI4P5K-A05–6* and *GbPI4P5K-A05-7* (**Supplementary Figure S4**). The results emphasized *PI4P5K* genes were highly conserved for gene quantity during polyploidization, while afterward same directional evolution for different allotetraploid cotton species.

### 3.4 Expression analysis of *PI4P5K* genes in *G. hirsutum*

**Figure 5:**
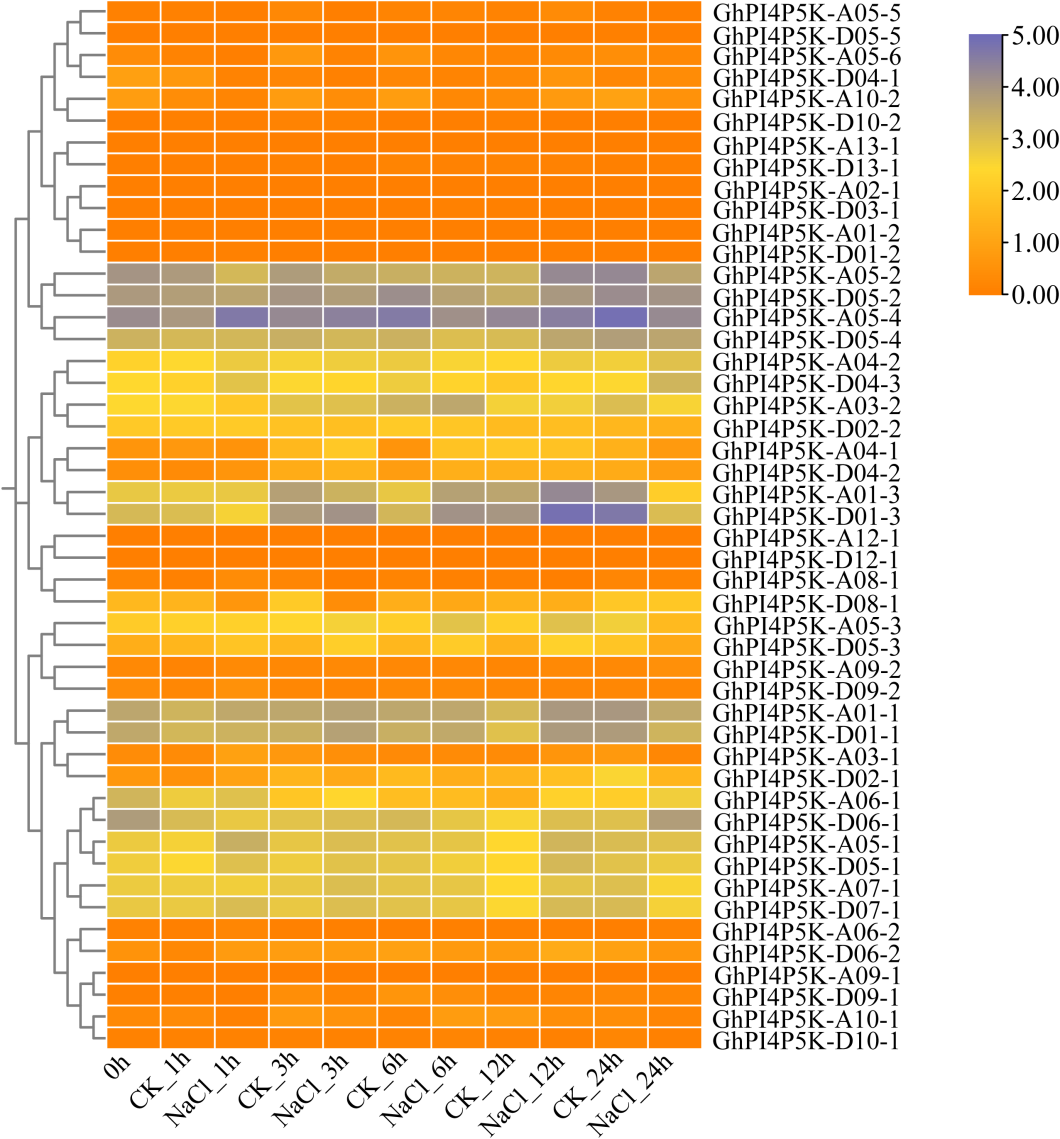
Expression analysis of the *PI4P5K* genes from *G. hirsutum* under salt stress based on transcriptome data. The heatmap color intensity represents the log2-transformed relative expression level; the color key indicates log2 values ranging from 0.00 (orange, lower expression) to 5.00 (purple, higher expression). CK: control group; 0h/1h/3h/6h/12h/24h: time points after NaCl treatment.

Gene expression patterns are usually related to gene function (Zhao et al., 2021). We investigated the expression patterns of *GhPI4P5K* genes in eleven tissues of cotton (Ovule, Fiber, Root, Stem, Leaf, Torus, Petal, Sepal, Epicalyx, Anther and Pistil). The results showed that most of the 48 selected *GhPI4P5K* genes exhibited tissue expression specificity (**Supplementary Figure S5**). For example, *GhPI4P5K-D05-2*, *GhPI4P5K-A05-2*, *GhPI4P5K-A01–1* and *GhPI4P5K-D01–1* had the highest expression level in ovule of 10 dpa (day post-anthesis), *GhPI4P5K-A03–1* and *GhPI4P5K-A03–2* in fiber of 20 dpa, *GhPI4P5K-A01–3* and *GhPI4P5K-D01–3* in root, and *GhPI4P5K*-*D09–2* in pistil. *PI4P5K* gene family plays an important role in plant growth, development, and stress response. So, we focused on the analysis of the expression patterns of *GhPI4P5K* genes under salt stress, and the results showed that the expression levels of many genes have changed with most exhibiting upregulation, among which five genes (*GhPI4P5K*-*A05*-*2*, *GhPI4P5K-A01-3*, *GhPI4P5K-D01-3*, *GhPI4P5K-A01-1*, *GhPI4P5K-D01-1*) were significantly upregulated, and their expression levels reached their highest after 12 h of salt treatment (**Figure 5**). In addition, *GhPI4P5K-A05–4* and *GhPI4P5K-A05–1* had the highest expression level at the time point of 3h after salt treatment, and *GhPI4P5K-A03–2* occurred at 6h later after salt stress (**Figure 5**). Moreover, *GhPI4P5K-D04–2* was a typical representative of the *PI4P5K* gene family in subgroup I in *G. hirsutum*, and its expression level had changed at 3 h, 6 h and 12 h obviously in upland cotton (**Figure 5**). Therefore, investigating the function of *GhPI4P5K-D04–2* gene under salt stress is representative.

### 3.5 *GhPI4P5K-D04–2* could increase salt stress tolerance in transgenic *Arabidopsis*

**Figure 6:**
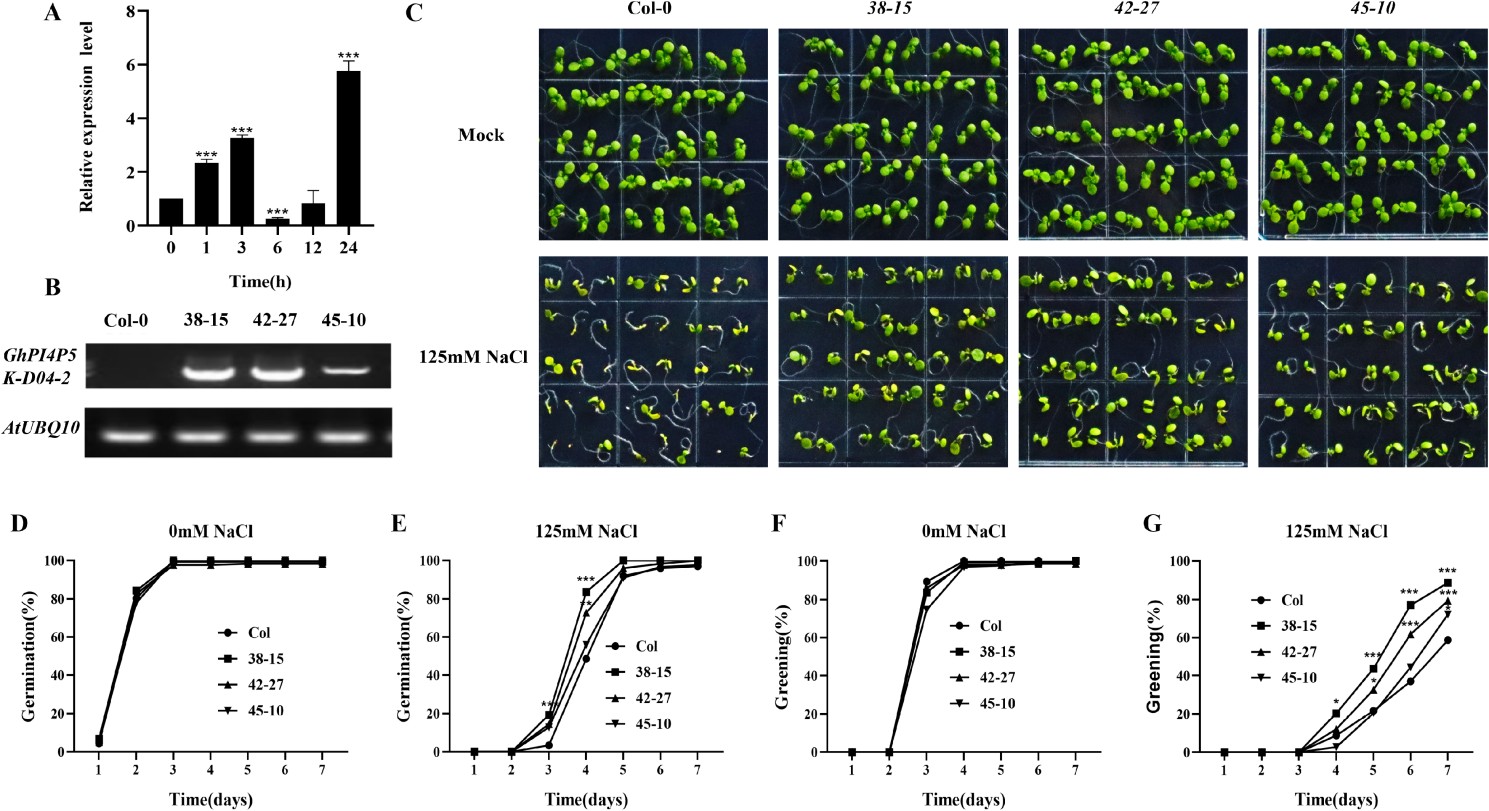
Phenotypic analysis of *GhPI4P5K-D04–2* overexpression *Arabidopsis* under salt stress conditions. **(A)** Relative expression level of *GhPI4P5K-D04–2* after salt stress treatment. **(B)** *GhPI4P5K-D04–2* detected in Col-0 and transgenic lines of *Arabidopsis*, with *AtUBQ10* as a control. **(C)** Place Col-0 and transgenic *Arabidopsis* on untreated MS medium and 125mM NaCl MS medium, and take photos after one week of growth. **(D)** Germination rates of Col-0 and transgenic *Arabidopsis* on MS medium. **(E)** Germination rates of Col-0 and transgenic *Arabidopsis* on MS medium supplemented with 125mM NaCl. **(F)** Greening rates of Col-0 and transgenic *Arabidopsis* on MS medium. **(G)** Greening rates of Col-0 and transgenic *Arabidopsis* on MS medium supplemented with 125mM NaCl. The error bars represent the means ± SD (n=3), perform significance analysis using t-test, *P < 0.05, **P < 0.01, ***P < 0.001.

To analyze the expression pattern of *GhPI4P5K-D04–2* in cotton, we subjected the three-leaf stage cotton to salt stress treatment and took its leaves for qRT-PCR analysis. The results showed that *GhPI4P5K-D04–2* showed a slight upregulation followed by a decrease and then upregulation trend, and the expression peak appeared 24 hours after salt stress treatment (**Figure 6A**). The above results indicated that *GhPI4P5K-D04–2* was involved in the response of plants to salt stress. To verify whether *GhPI4P5K-D04–2* could enhance plant salt tolerance, three transgenic *Arabidopsis* lines (38-15, 42–27 and 45-10) of T3 generation were obtained and checked by semi-quantitative PCR (**Figure 6B**). *Arabidopsis* seeds from three transgenic lines and the control Col-0 in the same culture dish were planted for growth. It was found that the transgenic strains exhibited stronger salt tolerance under treatment with 125mM NaCl (**Figure 6C**). In addition, the results of germination rate and greening rate showed that the transgenic strains were higher than those of Col-0 under salt stress (**Figures 6D–G**). All the results indicated that *GhPI4P5K-D04–2* positively regulated the salt resistance in transgenic *Arabidopsis*.

### 3.6 VIGS validation of *GhPI4P5K-D04–2* gene

**Figure 7:**
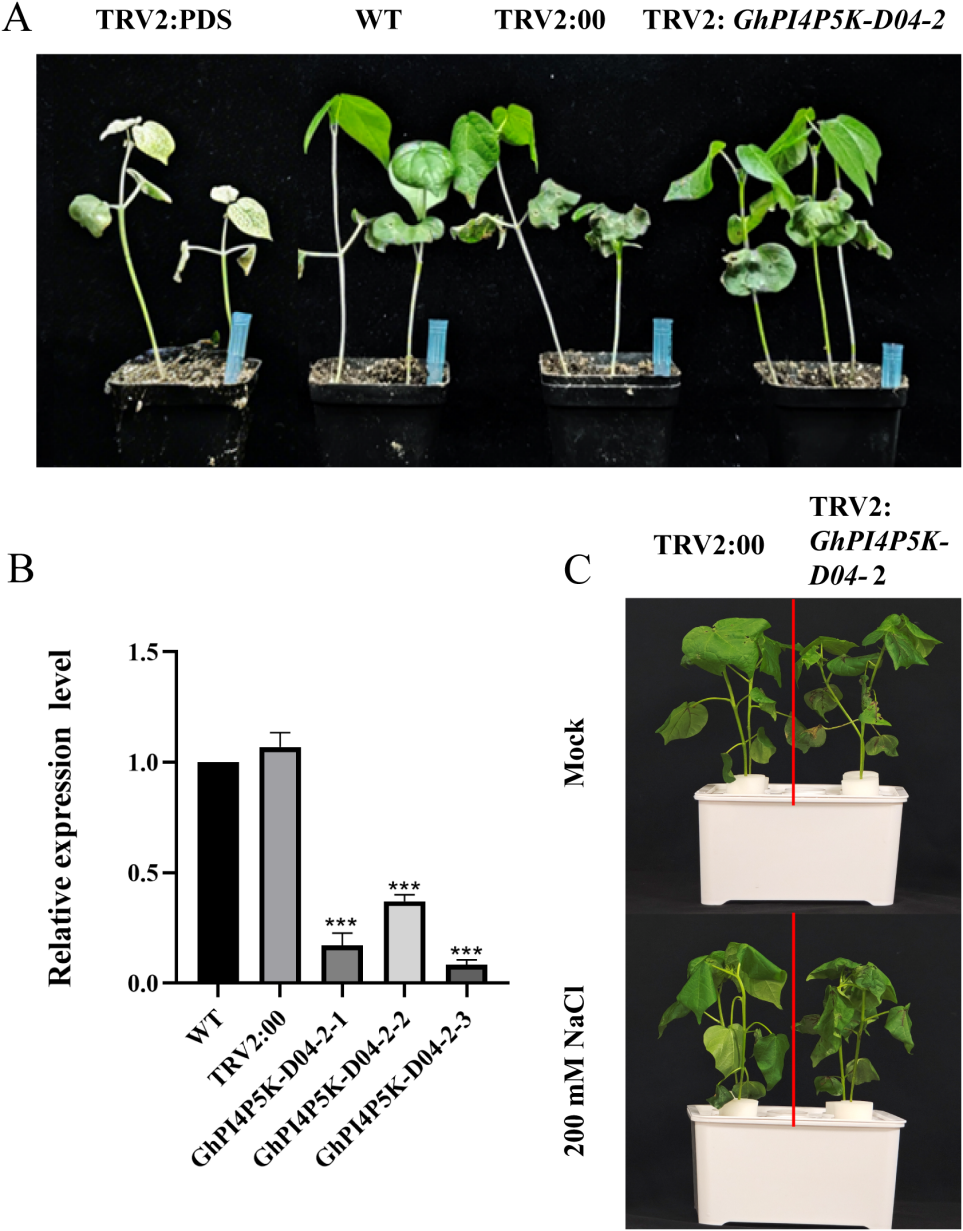
Virus-induced gene silencing of *GhPI4P5K-D04–2* and phenotype evaluation. **(A)** Feasibility assessment of VIGS technology system for cotton. **(B)** Assessment of the silencing efficiency of TRV2:00 and TRV2:*GhPI4P5K-D04-2*. The error bars represent the means ± SD (n=3), perform significance analysis using t-test, *** P < 0.001. **(C)** Leaf phenotype after treatment with 200 mM NaCl.

*PI4P5K* genes plays a key enzyme role in the inositol signal transduction system and has essential functions in plants in terms of growth, development, and stress responses. Therefore, we performed a virus-induced gene silencing (VIGS) experiment to verify its function under salt stress. After approximately 14 days of bacterial infection, TRV2:PDS cotton leaves showed an albino phenotype (**Figure 7A**). The silencing efficiency of *GhPI4P5K-D04–2* was evaluated using qPCR, and the results showed that the expression level of *GhPI4P5K-D04–2* gene in TRV2:*GhPI4P5K-D04–2* plants was significantly lower than that in TRV2:00 plants (**Figure 7B**). The above results indicated that the *GhPI4P5K-D04–2* gene was successfully silenced in cotton through the VIGS system. Plants of TRV2:*GhPI4P5K-D04–2* and TRV2:00 were subjected to 200 mM NaCl treatment. The results showed that there was no significant difference in growth status between TRV2:*GhPI4P5K-D04–2* and TRV2:00 plants under non-stressed conditions. Nevertheless, after approximately 48 h of exposure to 200 mM NaCl stress, TRV2:*GhPI4P5K-D04–2* plants exhibited more severe leaf wilting compared with TRV2:00 plants (**Figure 7C**). These results preliminarily indicated that silencing of *GhPI4P5K-D04–2* impaired the tolerance of plants to salt stress.

## 4 Discussion

Previous studies have shown that the phosphatidylinositol phosphate kinase gene family is widely present in living organisms (Saavedra et al., 2012). Phosphatidylinositol-4-phosphate 5-kinase (PI4P5K/PIP5K) stands as a vital enzyme within the phosphatidylinositol signaling pathway, exerting a substantial influence on plant growth, development, as well as the plant’s responses to both biotic and abiotic stresses (Mikami et al., 1998; Ma et al., 2004; Sharma et al., 2019). To date, the *PI4P5K* gene family has been investigated in diverse plants, including *Arabidopsis*, rice, wheat, ginkgo, soybean and tomato. Additionally, 11, 11, 64, 7, 22 and 21 PI4P5K family members have been identified in these plants, respectively (Zhang et al., 2020; Liu et al., 2021; Wang et al., 2023). However, there has been little reported about the cotton *PI4P5K* gene family. With the completion and continuous update of the cotton genome sequencing, it has become relatively easy to conduct research on gene families at the genomic level (Wang et al., 2012; Li et al., 2014, 2015; Hu et al., 2019; Wang et al., 2019). In this study, 146 *PI4P5K* genes were identified from the cotton genome and classified into three subgroups, which is consistent with the classification in wheat and tomato (Liu et al., 2021; Wang et al., 2023). While, this study is inconsistent with previous research on *PI4P5K* gene family in cotton. In the previous study, a total of 84 *PI4P5K* genes were obtained from four cotton species and classified into four subgroup (Qiao et al., 2024). It has 59 fewer genes than the PI4P5K family genes we screened, with 20 fewer in upland cotton (**Figures 1**, **2**). These results indicate that our research has expanded the number of genes in the PI4P5K family and predict the significance of this family in biological functions in cotton *G. hirsutum*. The structure of proteins and genes are fundamentally interconnected with the functional roles of genes. The analysis of gene structure and conserved domains showed that the cotton *PI4P5K* genes have a high degree of structural conservation, and all proteins contain PIPKc superfamily domains, which is consistent with previous research results in other plants (Heilmann and Heilmann, 2015). In subgroup III, in addition to the PIPKc superfamily domain, proteins also have domains that belong to chaperonin-like superfamily and FYVE-like-SF superfamily (**Figure 2**). These results suggest that proteins in subgroup III may have potential functions of binding to Zinc ion and phosphatidylinositol 3-phosphate (PI3P), and roles in normal cell growth and stress tolerance, which are presumably endowed by the FYVE-like-SF superfamily domain and chaperonin-like superfamily domain, respectively. The numbers of intron/exon might represent splicing variants and were used to classify genes (William Roy and Gilbert, 2006; Zhong et al., 2019). Based on the gene structure analysis, the number and structural characteristics of PI4P5K family genes in cotton exhibited significant variation across the three subgroups (**Supplementary Figures S1** and **S2**). Furthermore, although certain cotton *PI4P5K* genes share comparable coding sequence (CDS) numbers and lengths, their overall gene lengths differ markedly, suggesting substantial variation in intron lengths and different forms of gene splicing. The distribution of cotton *PI4P5K* genes in the A and D subgenome is highly similar, especially on chromosome 5, where the number of *PI4P5K* genes is the highest (**Figure 3**). This discovery indicates a strong collinearity between the A and D subgenome, and is consistent with the polyploid evolutionary history of cotton (Wendel and Cronn, 2003). The expression of *AtPIP5K1* is induced by drought, salinity, and abscisic acid stress, indicating its role in the transduction of water stress signal (Mikami et al., 1998). Kuroda report three genes of *PI4P5K* gene family involved in root growth under stress conditions (Kuroda et al., 2021). Moreover, the PI4P5K family genes in wheat play a role in male sterility induced by high temperatures (Liu et al., 2021). These reports indicate the roles in stress response of plant PI4P5K family genes.

Gene expression patterns offer critical insights for elucidating gene function. Therefore, we conducted a tissue expression pattern analysis of the PI4P5K family genes in *G. hirsutum*. The results showed that most of the genes in this family have specificity for tissue expression that suggest their important role in cotton growth and development. For cotton, the most important agronomic traits are fibers, which include ovulate development and fiber elongation. In this study, we found that *GhPI4P5K-A04–4* had the highest expression level in ovules at 0 dpa, and *GhPI4P5K-A01-1*, *GhPI4P5K-D01-1*, *GhPI4P5K-A05–2* and *GhPI4P5K-D05–2* at 10 dpa (**Supplementary Figure S5**). In addition, the expressions of *GhPI4P5K-A03–1* and *GhPI4P5K-A03–2* were significantly upregulated in fibers at 20 dpa. The above results suggested the significant role of the *PI4P5K* gene family in fiber development. Here, we analyzed the gene expression pattern of the PI4P5K family genes in *G. hirsutum* under salt stress, and many genes were upregulated, especially *GhPI4P5K-A01-1*, *GhPI4P5K-D01-1*, *GhPI4P5K-A05-1*, *GhPI4P5K-A01-3*, *GhPI4P5K-D01-3*, *GhPI4P5K-A03-2*, *GhPI4P5K*-*A05–2* and *GhPI4P5K-A05-4*, though the times when their expression levels reached their peak were different (**Figure 5**). These results indicated that the PI4P5K family genes in cotton have corresponding functions under salt stress condition. In addition to genes with significantly upregulated expression, some genes showed a moderate level of upregulation. Among them, *GhPI4P5K-D04–2* is a typical representative (**Figure 5**). Thus, we checked the expression pattern of *GhPI4P5K-D04–2* under salt stress and it showed a slight upregulation followed by a decrease and then significant upregulation trend (**Figure 6A**). To further study the function of *GhPI4P5K-D04–2* under salt stress, it was transformed into *Arabidopsis* and we obtained the overexpression transgenic lines. The seeds of *GhPI4P5K-D04–2* overexpression *Arabidopsis* were sown on MS medium with 125 mM NaCl and the results showed that *GhPI4P5K-D04–2* could enhance salt tolerance of transgenic *Arabidopsis* (**Figures 6B-G**). Furthermore, *GhPI4P5K-D04–2* knockdown cotton plants generated via virus-induced gene silencing (VIGS) displayed increased sensitivity to salt stress (**Figure 7**). The above results indicated that the *GhPI4P5K-D04–2* gene can indeed participate in the response to salt stress. However, the underlying molecular mechanism remains unclear, as direct experimental evidence is currently lacking. Potential regulatory pathways still need to be investigated in future functional studies.

In this study, we identified 146 *PI4P5K* genes from four cotton species and conducted systematical bioinformatics analysis. In addition, we further investigated the function of *GhPI4P5K-D04–2* in transgenic *Arabidopsis*. Overexpression of *GhPI4P5K-D04–2* can improve salt tolerance, germination rate, and greening rate of *Arabidopsis* under salt stress. Our research might provide some basis for the interpretation of *GhPI4P5K-D04–2* gene functions and potential gene resources for cotton salt stress resistance.

## Data Availability

The datasets presented in this study can be found in the article/**Supplementary Material**.
