# Supplementary material for: Genome-wide identification of the PI4P5K gene family in cotton and role of GhPI4P5K-D04-2 in salt stress tolerance
Source: Front Plant Sci. 2026 Feb 25;17:1750290. doi: 10.3389/fpls.2026.1750290 (PMC12975466; doi:10.3389/fpls.2026.1750290)
Supplement: Supplementary file 1 [file Supplementaryfile1.docx]

Supplementary Material

# Supplementary Figures and Tables

## Supplementary Figures


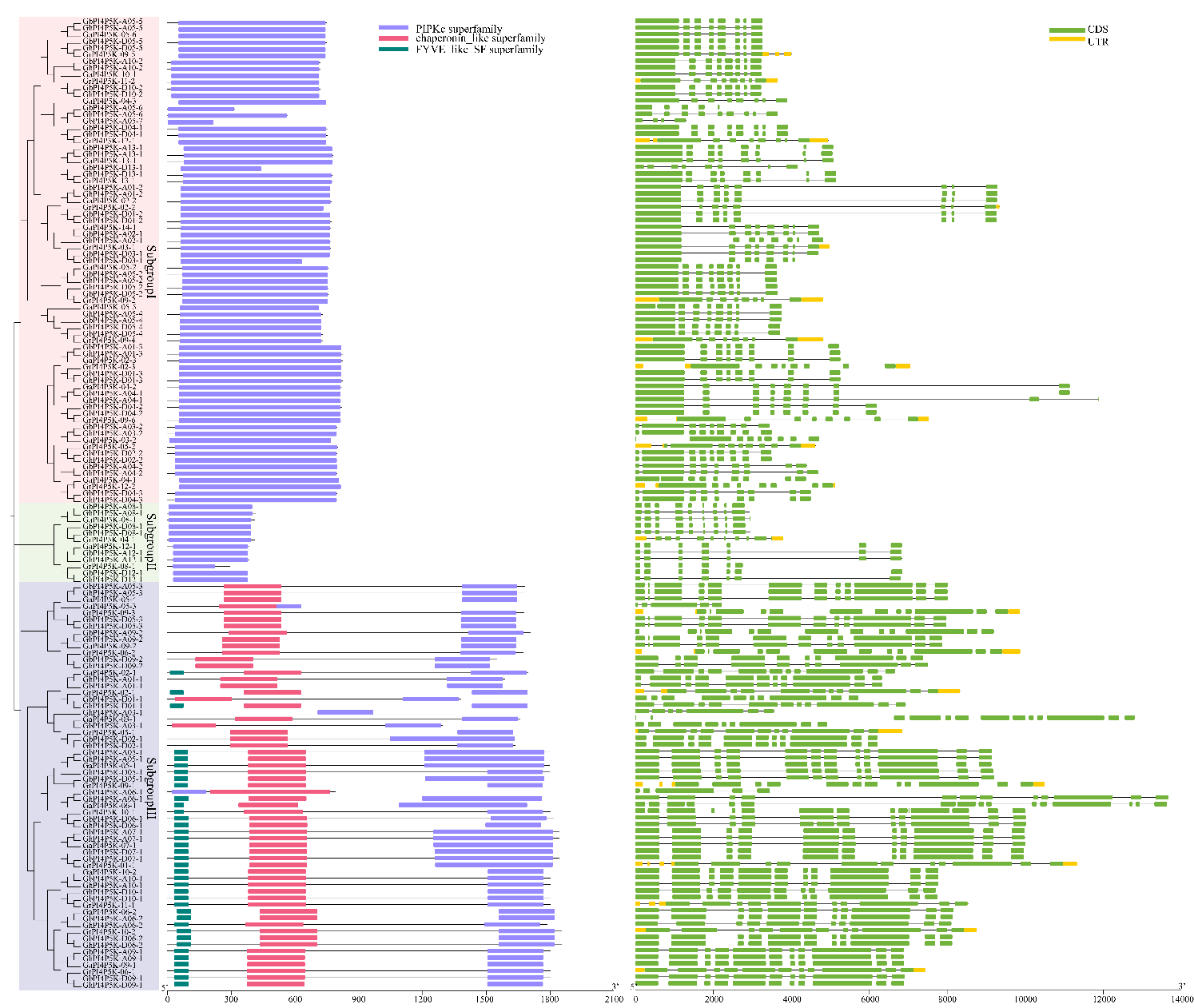


**Figure S1.** Analysis of phylogenetic tree, conserved domains and gene structure of PI4P5K family members in *G. arboreum*, *G. raimondii*, *G. barbadense* and *G. hirsutum*.


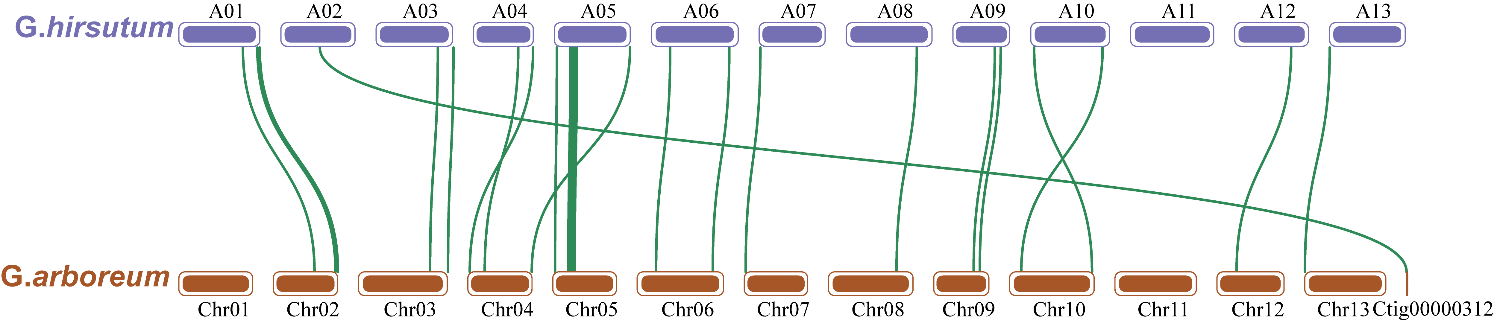


**Figure S2.** Collinearity relationship analysis of *PI4P5K* genes in subgenome A of *G. hirsutum* and *G. arboreum*.


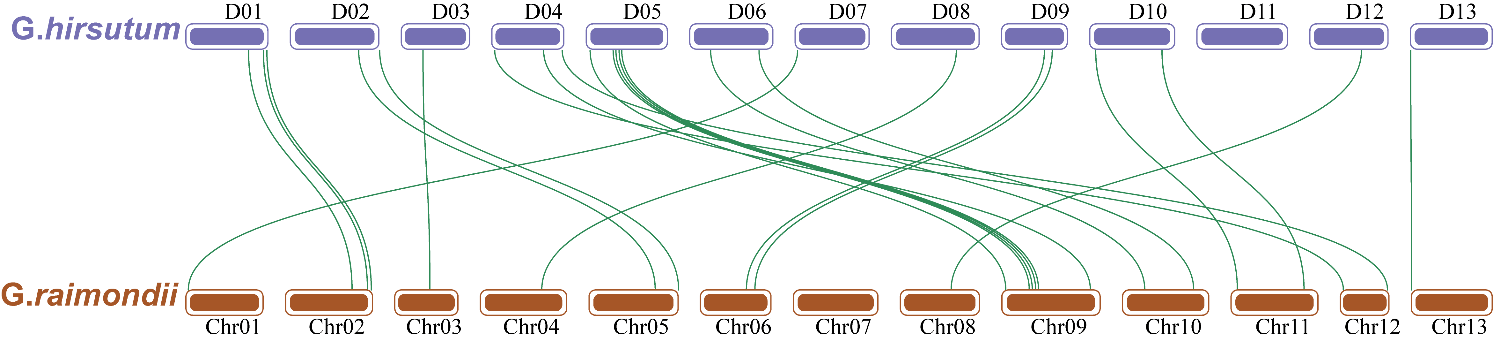


**Figure S3.** Collinearity relationship analysis of *PI4P5K* genes in subgenome D of *G. hirsutum* and *G. raimondii*.


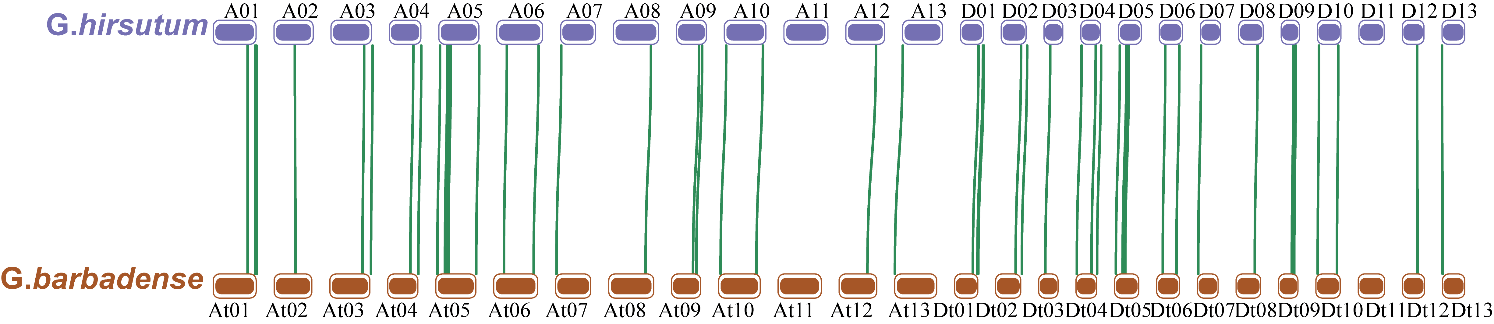


**Figure S4.** Collinearity relationship analysis of *PI4P5K* genes in *G. hirsutum* and *G. barbadense*.


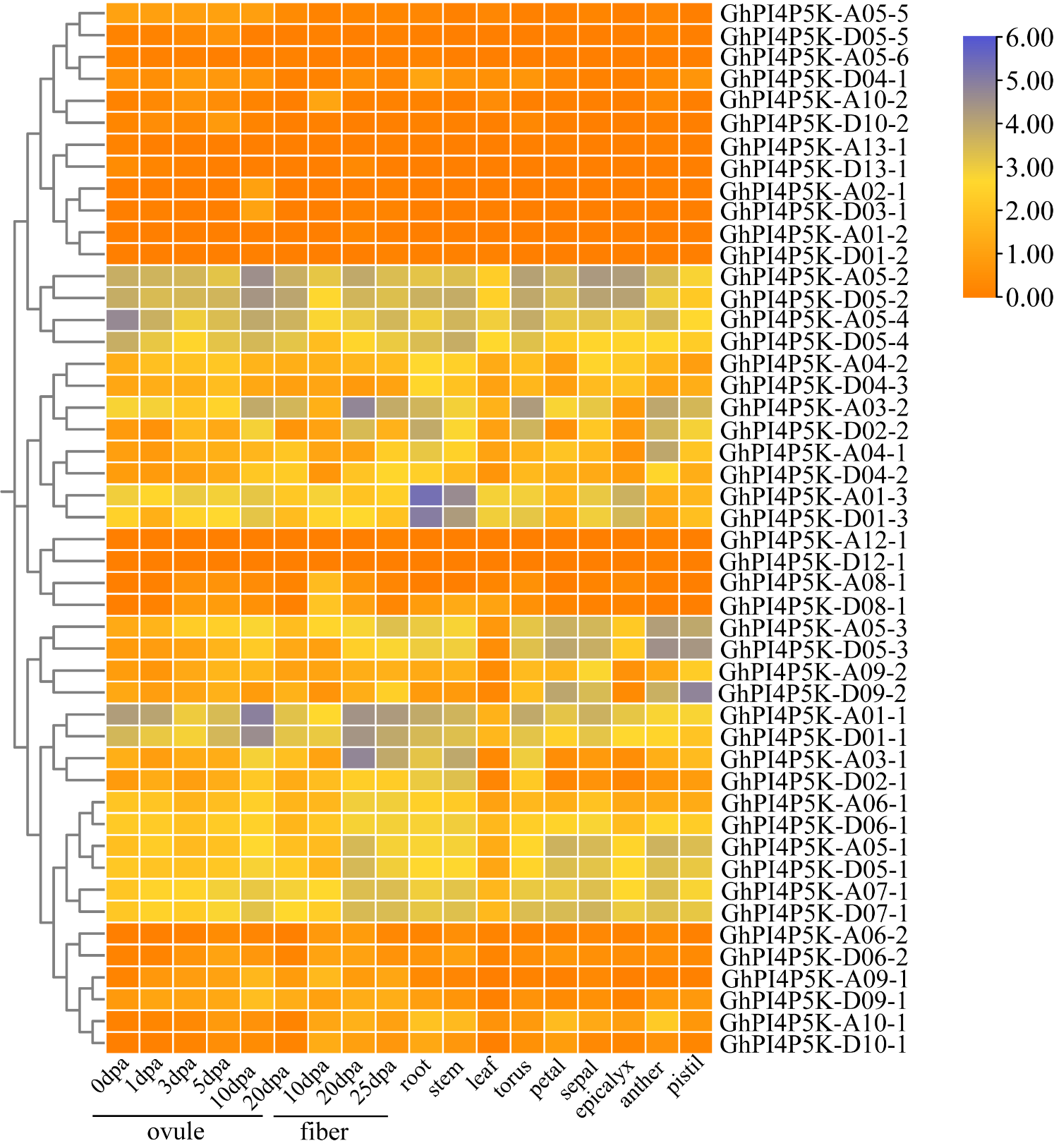


**Figure S5.** Expression analysis of the *PI4P5K* genes from *G. hirsutum* in different tissues.

| **Table S1.** *PI4P5K* gene family information in four cotton species | | | |
| --- | --- | --- | --- |
| Specie | Number | Gene name | Chromosome |
| *G. arboretum* | 25 | GaPI4P5K-02-1 | A02 |
|  |  | GaPI4P5K-02-2 | A02 |
|  |  | GaPI4P5K-02-3 | A02 |
|  |  | GaPI4P5K-03-1 | A03 |
|  |  | GaPI4P5K-03-2 | A03 |
|  |  | GaPI4P5K-04-1 | A04 |
|  |  | GaPI4P5K-04-2 | A04 |
|  |  | GaPI4P5K-04-3 | A04 |
|  |  | GaPI4P5K-05-1 | A05 |
|  |  | GaPI4P5K-05-2 | A05 |
|  |  | GaPI4P5K-05-3 | A05 |
|  |  | GaPI4P5K-05-4 | A05 |
|  |  | GaPI4P5K-05-5 | A05 |
|  |  | GaPI4P5K-05-6 | A05 |
|  |  | GaPI4P5K-06-1 | A06 |
|  |  | GaPI4P5K-06-2 | A06 |
|  |  | GaPI4P5K-07-1 | A07 |
|  |  | GaPI4P5K-08-1 | A08 |
|  |  | GaPI4P5K-09-1 | A09 |
|  |  | GaPI4P5K-09-2 | A09 |
|  |  | GaPI4P5K-10-1 | A10 |
|  |  | GaPI4P5K-10-2 | A10 |
|  |  | GaPI4P5K-12-1 | A12 |
|  |  | GaPI4P5K-13-1 | A13 |
|  |  | GaPI4P5K-14-1 | A14 |
| *G*. *raimondii* | 24 | GrPI4P5K-01-1 | D01 |
|  |  | GrPI4P5K-02-1 | D02 |
|  |  | GrPI4P5K-02-2 | D02 |
|  |  | GrPI4P5K-02-3 | D02 |
|  |  | GrPI4P5K-03-1 | D03 |
|  |  | GrPI4P5K-04-1 | D04 |
|  |  | GrPI4P5K-05-1 | D05 |
|  |  | GrPI4P5K-05-2 | D05 |
|  |  | GrPI4P5K-06-1 | D06 |
|  |  | GrPI4P5K-06-2 | D06 |
|  |  | GrPI4P5K-08-1 | D08 |
|  |  | GrPI4P5K-09-1 | D09 |
|  |  | GrPI4P5K-09-2 | D09 |
|  |  | GrPI4P5K-09-3 | D09 |
|  |  | GrPI4P5K-09-4 | D09 |
|  |  | GrPI4P5K-09-5 | D09 |
|  |  | GrPI4P5K-09-6 | D09 |
|  |  | GrPI4P5K-10-1 | D10 |
|  |  | GrPI4P5K-10-2 | D10 |
|  |  | GrPI4P5K-11-1 | D11 |
|  |  | GrPI4P5K-11-2 | D11 |
|  |  | GrPI4P5K-12-1 | D12 |
|  |  | GrPI4P5K-12-2 | D12 |
|  |  | GrPI4P5K-13-1 | D13 |
| *G*. *barbadense* | 49 | GbPI4P5K-A01-1 | A01 |
|  |  | GbPI4P5K-A01-2 | A01 |
|  |  | GbPI4P5K-A01-3 | A01 |
|  |  | GbPI4P5K-A02-1 | A02 |
|  |  | GbPI4P5K-A03-1 | A03 |
|  |  | GbPI4P5K-A03-2 | A03 |
|  |  | GbPI4P5K-A04-1 | A04 |
|  |  | GbPI4P5K-A04-2 | A04 |
|  |  | GbPI4P5K-A05-1 | A05 |
|  |  | GbPI4P5K-A05-2 | A05 |
|  |  | GbPI4P5K-A05-3 | A05 |
|  |  | GbPI4P5K-A05-4 | A05 |
|  |  | GbPI4P5K-A05-5 | A05 |
|  |  | GbPI4P5K-A05-6 | A05 |
|  |  | GbPI4P5K-A05-7 | A05 |
|  |  | GbPI4P5K-A06-1 | A06 |
|  |  | GbPI4P5K-A06-2 | A06 |
|  |  | GbPI4P5K-A07-1 | A07 |
|  |  | GbPI4P5K-A08-1 | A08 |
|  |  | GbPI4P5K-A09-1 | A09 |
|  |  | GbPI4P5K-A09-2 | A09 |
|  |  | GbPI4P5K-A10-1 | A10 |
|  |  | GbPI4P5K-A10-2 | A10 |
|  |  | GbPI4P5K-A12-1 | A12 |
|  |  | GbPI4P5K-A13-1 | A13 |
|  |  | GbPI4P5K-D01-1 | D01 |
|  |  | GbPI4P5K-D01-2 | D01 |
|  |  | GbPI4P5K-D01-3 | D01 |
|  |  | GbPI4P5K-D02-1 | D02 |
|  |  | GbPI4P5K-D02-2 | D02 |
|  |  | GbPI4P5K-D03-1 | D03 |
|  |  | GbPI4P5K-D04-1 | D04 |
|  |  | GbPI4P5K-D04-2 | D04 |
|  |  | GbPI4P5K-D04-3 | D04 |
|  |  | GbPI4P5K-D05-1 | D05 |
|  |  | GbPI4P5K-D05-2 | D05 |
|  |  | GbPI4P5K-D05-3 | D05 |
|  |  | GbPI4P5K-D05-4 | D05 |
|  |  | GbPI4P5K-D05-5 | D05 |
|  |  | GbPI4P5K-D06-1 | D06 |
|  |  | GbPI4P5K-D06-2 | D06 |
|  |  | GbPI4P5K-D07-1 | D07 |
|  |  | GbPI4P5K-D08-1 | D08 |
|  |  | GbPI4P5K-D09-1 | D09 |
|  |  | GbPI4P5K-D09-2 | D09 |
|  |  | GbPI4P5K-D10-1 | D10 |
|  |  | GbPI4P5K-D10-2 | D10 |
|  |  | GbPI4P5K-D12-1 | D12 |
|  |  | GbPI4P5K-D13-1 | D13 |
| *G*. *hirsutum* | 48 | GhPI4P5K-A01-1 | A01 |
|  |  | GhPI4P5K-A01-2 | A01 |
|  |  | GhPI4P5K-A01-3 | A01 |
|  |  | GhPI4P5K-A02-1 | A02 |
|  |  | GhPI4P5K-A03-1 | A03 |
|  |  | GhPI4P5K-A03-2 | A03 |
|  |  | GhPI4P5K-A04-1 | A04 |
|  |  | GhPI4P5K-A04-2 | A04 |
|  |  | GhPI4P5K-A05-1 | A05 |
|  |  | GhPI4P5K-A05-2 | A05 |
|  |  | GhPI4P5K-A05-3 | A05 |
|  |  | GhPI4P5K-A05-4 | A05 |
|  |  | GhPI4P5K-A05-5 | A05 |
|  |  | GhPI4P5K-A05-6 | A05 |
|  |  | GhPI4P5K-A06-1 | A06 |
|  |  | GhPI4P5K-A06-2 | A06 |
|  |  | GhPI4P5K-A07-1 | A07 |
|  |  | GhPI4P5K-A08-1 | A08 |
|  |  | GhPI4P5K-A09-1 | A09 |
|  |  | GhPI4P5K-A09-2 | A09 |
|  |  | GhPI4P5K-A10-1 | A10 |
|  |  | GhPI4P5K-A10-2 | A10 |
|  |  | GhPI4P5K-A12-1 | A12 |
|  |  | GhPI4P5K-A13-1 | A13 |
|  |  | GhPI4P5K-D01-1 | D01 |
|  |  | GhPI4P5K-D01-2 | D01 |
|  |  | GhPI4P5K-D01-3 | D01 |
|  |  | GhPI4P5K-D02-1 | D02 |
|  |  | GhPI4P5K-D02-2 | D02 |
|  |  | GhPI4P5K-D03-1 | D03 |
|  |  | GhPI4P5K-D04-1 | D04 |
|  |  | GhPI4P5K-D04-2 | D04 |
|  |  | GhPI4P5K-D04-3 | D04 |
|  |  | GhPI4P5K-D05-1 | D05 |
|  |  | GhPI4P5K-D05-2 | D05 |
|  |  | GhPI4P5K-D05-3 | D05 |
|  |  | GhPI4P5K-D05-4 | D05 |
|  |  | GhPI4P5K-D05-5 | D05 |
|  |  | GhPI4P5K-D06-1 | D06 |
|  |  | GhPI4P5K-D06-2 | D06 |
|  |  | GhPI4P5K-D07-1 | D07 |
|  |  | GhPI4P5K-D08-1 | D08 |
|  |  | GhPI4P5K-D09-1 | D09 |
|  |  | GhPI4P5K-D09-2 | D09 |
|  |  | GhPI4P5K-D10-1 | D10 |
|  |  | GhPI4P5K-D10-2 | D10 |
|  |  | GhPI4P5K-D12-1 | D12 |
|  |  | GhPI4P5K-D13-1 | D13 |
